# Vaccinia Virus LC16m8∆ as a Vaccine Vector for Clinical Applications

**DOI:** 10.3390/vaccines2040755

**Published:** 2014-10-17

**Authors:** Minoru Kidokoro, Hisatoshi Shida

**Affiliations:** 1 Department of Virology III, National Institute of Infectious Diseases, 4-7-1 Gakuen, Musashimurayama-shi, Tokyo 208-0011, Japan; E-Mail: kidokoro@nih.go.jp; 2 Institute for Genetic Medicine, Hokkaido University, Kita-15, Nishi-7, Kita-ku, Sapporo 060-0815, Japan

**Keywords:** LC16m8∆, LC16m8, vaccinia virus, reversion, *B5R*, MVA, DIs, SIV, HIV

## Abstract

The LC16m8 strain of vaccinia virus, the active ingredient in the Japanese smallpox vaccine, was derived from the Lister/Elstree strain. LC16m8 is replication-competent and has been administered to over 100,000 infants and 3,000 adults with no serious adverse reactions. Despite this outstanding safety profile, the occurrence of spontaneously-generated large plaque-forming virulent LC16m8 revertants following passage in cell culture is a major drawback. We identified the gene responsible for the reversion and deleted the gene (*B5R*) from LC16m8 to derive LC16m8Δ. LC16m8∆ is non-pathogenic in immunodeficient severe combined immunodeficiency (SCID) mice, genetically-stable and does not reverse to a large-plaque phenotype upon passage in cell culture, even under conditions in which most LC16m8 populations are replaced by revertants. Moreover, LC16m8∆ is >500-fold more effective than the non-replicating vaccinia virus (VV), Modified Vaccinia Ankara (MVA), at inducing murine immune responses against pathogenic VV. LC16m8∆, which expresses the SIV *gag* gene, also induced anti-Gag CD8^+^ T-cells more efficiently than MVA and another non-replicating VV, Dairen I minute-pock variants (DIs). Moreover, LC16m8∆ expressing HIV-1 Env in combination with a Sendai virus vector induced the production of anti-Env antibodies and CD8^+^ T-cells. Thus, the safety and efficacy of LC16m8∆ mean that it represents an outstanding platform for the development of human vaccine vectors.

## 1. Introduction

### 1.1. First-Generation Smallpox Vaccines

Smallpox was eradicated worldwide in the 1970s [1,2]. However, serious public health concerns due to the threat of bioterrorism [3] and natural outbreaks of monkeypox [4,5] at the start of the 21st century have highlighted the necessity for a vaccinia virus (VV)-based smallpox vaccine. Existing vaccine stockpiles have not been updated since the 1970s; because these early vaccines are lymph-derived vaccines produced by propagating vaccine viruses in the skin of animals (*i.e.*, first-generation vaccines (Table 1)), they do not meet good manufacturing practice (GMP) standards [6,7,8]. Therefore, they are at risk for adventitious microbial contamination. Moreover, these vaccines occasionally caused serious adverse effects (e.g., progressive vaccinia, eczema vaccinatum and post-vaccinial encephalitis) due to the pathogenicity of the vaccine viruses used [9,10].

**Table 1:** Smallpox vaccines and candidate vaccines classified according to generation.

Generation	Product	Platform	Parental Strain
First-generation	Lister/Elstree	Lymph-derived	Lister/Elstree
	Dryvax	Lymph-derived	NYCBH ^a^
	Ikeda	Lymph-derived	Ikeda
	Dairen I	Lymph-derived	Dairen I
Second-generation	ACAM1000	Clonal virus grown in MRC-5 cells	Dryvax
	ACAM2000	Clonal virus grown in Vero cells	ACAM1000
	Elstree-BN	Lister/Elstree lymph-derived virus passaged in CEF ^b^	Lister/Elstree
	CCSV	NYCBH lymph-derived virus passaged in MRC-5 cells	NYCBH
Third-generation	LC16m8 ^c^	Minute-pock-forming, temperature-sensitive variant virus	Lister/Elstree
	IMVAMUNE (MVA ^d^)	MVA571 additionally passaged in CEF	MVA571
	DIs ^e^	Minute-pock-forming variant virus passaged in eggs	Dairen I
Fourth-generation	LC16m8∆	Derived from LC16m8 by deleting the *B5R* gene	LC16m8
	NYVAC	Attenuated clonal Copenhagen strain generated by deleting 18 non-essential genes	Copenhagen

^a^ New York City Board of Health; ^b^ chicken embryo fibroblast; ^c^ Lister Clone 16m8; ^d^ Modified Vaccinia Ankara; ^e^ Dairen I minute-pock variants.

### 1.2. Second-Generation Vaccines 

To address the issues outlined above, much effort has gone into developing safer smallpox vaccine candidates. Some studies aimed to generate vaccines using a sterile cell culture technique to reduce the risk of contamination by adventitious agents (second-generation vaccines) (see Table 1) [11]. For example, ACAM1000 [12,13] was propagated in MRC-5 cells (diploid human lung fibroblasts) using a single clone VV isolated from a Dryvax calf lymph vaccine (manufactured by Wyeth Laboratories using New York City Board of Health (NYCBH)). ACAM2000 was prepared in Vero cells under serum-free conditions using ACAM1000 as the seed virus [13,14]. The cell-cultured smallpox vaccine (CCSV), which was derived from a plaque-purified NYCBH strain, was also prepared in MRC-5 cells [15]. The Elstree-BN vaccine was manufactured in chicken embryo fibroblasts (CEF) using the Lister/Elstree (Lister) strain, which was widely used as a lymph-derived vaccine in Europe, Africa and Asia during the global smallpox eradication campaign [16]. The manufacturing of vaccines in cell culture reduced the risk of vaccine contamination by extraneous agents. However, because second-generation vaccines were manufactured using first-generation vaccines or their isolates as seed viruses, their safety profiles were equivalent to those of the original lymph-derived vaccines, *i.e.*, they caused the same adverse events [13,15].

### 1.3. Third-Generation Vaccines

As the global smallpox eradication campaign progressed and the risk of contracting smallpox infections diminished, developed countries began to raise concerns about the side effects associated with lymph-derived smallpox vaccines. This triggered new research to develop alternative attenuated vaccines (third-generation vaccines), such as the Modified Vaccinia Ankara (MVA) [17,18], Dairen I minute-pock variants (DIs) [19] and Lister Clone 16m8 (LC16m8) [20,21,22,23]. The main method used to attenuate the VVs was serial passage in primary cell culture or eggs.

MVA was attenuated by serial passage of the chorioallantois VV Ankara strain in CEF (>570 times). This resulted in the loss of approximately 15% of its genome, including host range-related genes, such as *K1L*, and some immunomodulatory genes, thereby generating a phenotype that is unable to replicate in most mammalian cells [24,25,26]. MVA, which shows an extremely attenuated phenotype in animals and humans [27], was administered to about 120,000 individuals in Western Germany and Turkey during the global eradication campaign with no apparent side effects [28]. Although its ability to protect against smallpox infection was not proven at that time, the need for a smallpox vaccine that was safe for use in immunocompromised individuals (including AIDS patients, patients treated with chemotherapy and transplant recipients) meant that MVA was examined in a number of clinical trials [29,30,31,32,33]. Data from these clinical trials and some animal experiments suggest that although MVA-derived vaccines have an extremely good safety profile; they are less immunogenic than replication-competent VVs, such as Dryvax, LC16m8 and LC16m8∆ [30,34,35,36]. For example, data from animal models show that multiple and 1–2 log higher doses of MVA are required to elicit antibody titers comparable with those elicited by replication-competent VVs [34,35,36].

The DIs strain originates from the Dairen I strain, a smallpox vaccine strain in Japan, and was isolated as a small-sized pock forming variant on chick chorioallantoic membrane (CAM) after 13 passages in one-day-old eggs [19]. As is the case with MVA, DIs harbors a large deletion within the left terminal region of the genome, which contains the host range genes, *K1L* and *C7L*, and the immunomodulatory gene, *K3L* [37]; consequently, DIs lacks the ability to replicate in a number of mammalian cell types. Although DIs showed a good safety profile when tested in field trials involving 200 Japanese children, it was not adopted as a smallpox vaccine, because it was less immunogenic than Lister Clone 16 (LC16).

Concerns regarding the side effects of first-generation smallpox vaccines, such as Ikeda, Dairen I and Lister were becoming a problem in Japan during the 1970s. In response to demands for a safer (but still effective) vaccine, the Chiba Serum Institute developed a highly attenuated strain, called LC16m8 [20,23]. LC16m8, which forms minute pocks on the CAM of embryonated eggs, was isolated from the Lister (Lister original, LO) strain via intermediate strains, such as LC16 and its derivative, LC16mO [23,38]. Tests in rabbit and monkey models showed that LC16m8 was markedly less neurovirulent than first-generation vaccine strains, such as LO and Dryvax; indeed, its virulence was comparable with that of replication-defective DIs [21,22,23,39]. Moreover, LC16m8 induced a much weaker dermal reaction in rabbits and humans and showed a lower rate of febrile reactions than LC16mO (a direct parent of LC16m8) in clinical trials [23,40]. LC16m8 was administrated to approximately 100,000 infants without any serious adverse reactions and proved to be as immunogenic as the parental LO strain [23,40]. Therefore, LC16m8 was adopted as the favored vaccine strain in Japan [40].

### 1.4. Fourth-Generation Vaccines 

A number of novel attenuation approaches involving direct modification of the VV genome using genetic engineering techniques were used to develop highly attenuated VV strains (fourth-generation vaccines), such as NYVAC and LC16m8∆ [6,34,41,42,43,44,45,46]. These methods replaced classical attenuation methods based on serial passage in primary cell cultures or eggs. NYVAC was derived from the Copenhagen VV vaccine strain by deleting 18 non-essential genes, which include *C7L* and *K1L*, the host range genes; the thymidine kinase gene, a gene related to viral DNA synthesis and the *I4L* gene encoding the large subunit of ribonucleotide reductase. Thus, NYVAC shows very restricted replication in mammalian cells and a highly attenuated phenotype in animals [41]. However, since the replication of NYVAC in non-permissive mammal cells is arrested at an early stage [47] (as is the case for avipoxviruses, such as canary poxvirus and fowl poxvirus), it elicits weaker immune responses than MVA or replication-competent VVs [48].

LC16m8∆ should be categorized as a fourth-generation vaccine, because it was obtained from the parental smallpox vaccine strain (LC16m8) by deleting the *B5R* gene, which is responsible for the reversion of LC16m8. Consequently, it shows good genetic stability with very little (if any) reversion; however, it retains its ability to replicate in mammalian cells [34].

## 2. LC16m8 and *B5R*

Takahashi-Nishimaki *et al.* first identified the VV *B5R* gene, which is responsible for large-plaque formation and replication in Vero cells, during the course of investigating the mechanism of attenuation to generate LC16m8 [49]. LC16m8 harbors a frameshift mutation due to a single base deletion in the middle of its open reading frame (ORF); this mutation results in the loss of *B5R* function. *B5R* encodes a 42-kDa glycoprotein (B5 protein), which is involved in packaging the intracellular mature virion (IMV) within the trans-Golgi membrane or endosomal cisternae to form an intracellular enveloped virion (IEV) [50,51,52]. The IEV is transported along microtubules to the cell periphery [53,54], where it adheres to the cell membrane as a cell-associated enveloped virion (CEV). The B5 protein, in cooperation with the A36 and A33 proteins, also participates in the Src kinase-dependent formation of actin-containing microvilli and the subsequent release of the CEV from the cell surface to form an extracellular enveloped virion (EEV) [55,56]. Despite the relative paucity of whole progeny virions, EEVs play an important role in dissemination within the host [57]. Since anti-B5 antibodies neutralize EEV, *B5R-*expressing VV has been proposed as an effective smallpox vaccine [50,58,59,60,61].

When generating and performing basic research on LC16m8, we found that the vaccine spontaneously reverted to large-plaque-forming clones (LPCs) [34]. Thus, we were concerned that LPC contamination might ruin the safety profile of a future LC16m8 vaccine. Therefore, we investigated the molecular mechanism(s) underlying the reversion, with a focus on the *B5R* gene associated with the formation of large plaques [49]. 

We isolated three LPC clones from a vaccine stock of LC16m8 and examined their phenotypes in terms of plaque size, dermal reactions in rabbits and pathogenicity in severe combined immunodeficiency (SCID) mice; these phenotypes were compared with those of LC16m8 and the parental virus LC16mO, which retains a fully-functional *B5R* gene. All three LPC viruses showed phenotypes similar to that of LC16mO, resulting in better growth in cell culture and greater virulence in SCID mice than LC16m8 [34]. As expected, sequencing the *B5R* in these LPCs revealed that the *B5R* ORF contained a single base insertion upstream of the nucleotide that was deleted from the LC16m8 *B5R.* It is noteworthy that the nucleotide insertion site in the LPC *B5R* ORF was different in each of the three clones, even though they originated from the same viral stock, which was prepared through only seven passages after the LC16m8 cloning. These results strongly suggest that the reversion of LC16m8 is a multi-clonal event and may occur frequently.

## 3. LC16m8Δ

### 3.1. Safety Profile

**Figure 1:**
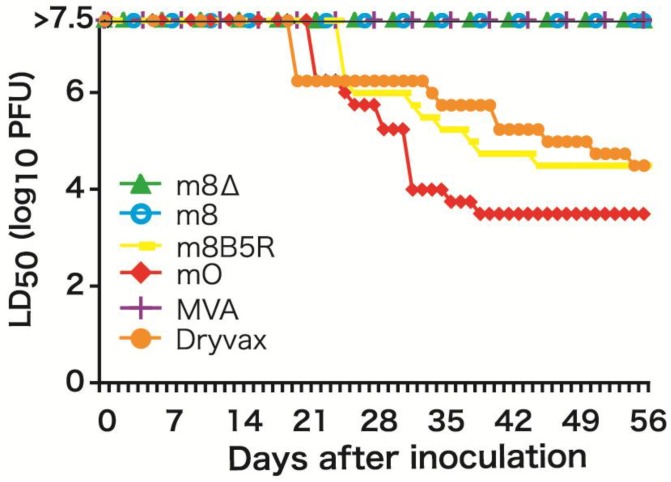
Pathogenicity of *B5R*-defective viruses in severe combined immunodeficiency (SCID) mice. Figure modified from Kidokoro *et al.* [34].

To prevent the generation of LC16m8 revertants, we decided to delete the entire *B5R* gene from the LC16m8 genome by homologous recombination to yield LC16m8∆ [34]. The phenotype of LC16m8∆ (plaque size and dermal reaction in rabbits) was similar to that of LC16m8. Intraperitoneal (i.p.) injection of 10^7^ PFU of LC16m8∆ (a dose three logs higher than that needed to elicit protective immunity in BALB/c mice) did not cause any symptoms in SCID mice over an eight-week period (Figure 1). MVA and plaque-purified LC16m8 (which contains a very low level of revertants) were also nonpathogenic; however, LC16mO and m8*B5R* (a derivative constructed by reintroducing the intact *B5R* gene into LC16m8) caused severe rashes and death in SCID mice, even when administered at a dose two logs lower than LC16m8∆ (Figure 1). 

**Figure 2:**
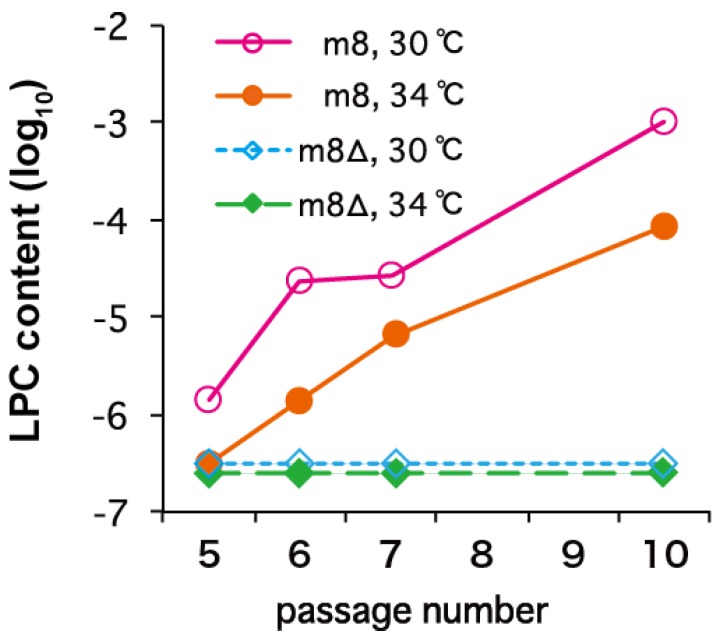
Genetic stability of LC16m8∆ and LC16m8 upon serial passage in primary rabbit kidney cells at different temperatures (30 °C or 34 °C). Figure modified from Kidokoro *et al.* [34]. LPC, large-plaque-forming clone.

The genetic stability of LC16m8∆ was evaluated by serial passage in primary rabbit kidney (PRK) cells, which were used to generate the LC16m8 vaccine. No detectable LPCs emerged from LC16m8∆ under any of the test conditions, including those used in vaccine production (passage in PRK cells at 30 °C). By contrast, LPCs emerged from LC16m8 that was plaque-purified immediately before testing (Figure 2). It should be noted that once LPCs appeared in the cultures, the LPCs:LC16m8 ratio increased rapidly with the passage number (Figure 2).

### 3.2. Immunogenicity

**Figure 3:**
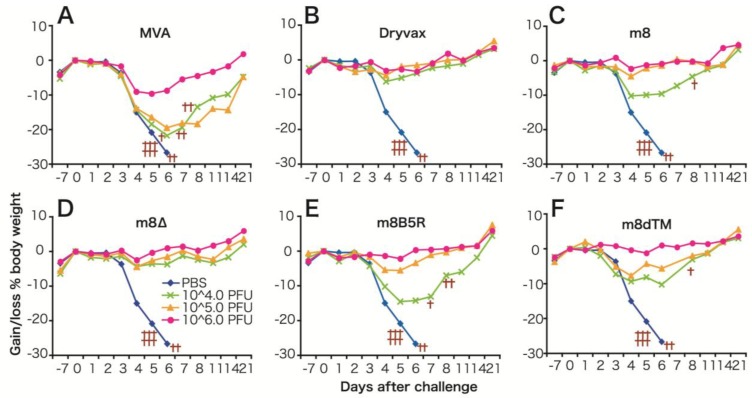
(**A**) Protective immune responses induced by m8∆ and derivative viruses in mice. (**A**–**F**) Average body weight of mice immunized (intramuscularly) with (10^4^–10^6^ PFU) vaccinia viruses (VVs) and then challenged intranasally with the Western Reserve (WR) strain. Crosses denote mice that either died or were sacrificed because they lost >30% of their body weight. Figure modified from Kidokoro *et al.* [34].

The protective immune response elicited by LC16m8∆ was compared with that elicited by Dryvax, MVA, LC16m8 and LC16m8 derivatives (m8*B5R* and m8dTM, both of which express the B5 ectodomain at high levels) in a mouse model. This model, in which the immunized mice are challenged with a highly pathogenic VV (the Western Reserve (WR) strain), is one of the most popular methods of evaluating the efficacy of smallpox vaccines [62] (Figure 3). We immunized each group of mice with a single dose (10^4^, 10^5^ or 10^6^ PFU) of each VV via the intramuscular (i.m.) route. We found that the level of protective immunity elicited by LCm8∆ was comparable with that elicited by Dryvax and superior to that elicited by MVA. For example, the minimal dose (10^4^ PFU) of LC16m8∆ or Dryvax fully protected mice from lethal infection with WR, whereas mice immunized with MVA, LC16m8, m8*B5R* or m8dTM, lost weight and, in some cases, died. The maximum dose (10^6^ PFU) of MVA resulted in prominent weight loss after WR challenge. It is noteworthy that immunization with LCm8∆ was more efficient than that with m8*B5R* or m8dTM when compared at their minimal dose. In particular, m8*B5R* was significantly inferior to LC16m8∆ (*t*-test, *p* = 0.005). These results suggest that *B5R* does not play a major role in eliciting protective immune responses in these mice. In addition, LC16m8∆ elicited protective immune responses in cynomolgus monkeys and fully protected them against lethal infection with monkeypox virus [63]. Taken together, these data suggest that LC16m8∆ is as effective as the first-generation smallpox vaccine, Dryvax. Although several studies report that the B5 protein is the major target of EEV-neutralizing antibodies, which are significant for protection against smallpox infection, immunization with B5-deficient vaccine viruses protects animals against lethal challenge by pathogenic orthopoxviruses [58,64,65,66,67]. In addition, some reports show that smallpox vaccines do not always induce anti-B5 antibodies, and antibody response profiles against each viral protein are highly heterologous in humans [68,69,70]. They also concluded that the key to inducing a strong neutralizing antibody response is to elicit antibodies that recognize multiple viral proteins; these antibodies then act synergistically to provide better protection.

## 4. LC16m8∆ as a Vehicle for Expressing Foreign Genes

VV has been widely used as a vector for expressing foreign genes, because it has many excellent properties: high expression efficiency, a broad host range, a very large capacity for accepting foreign genes, heat stability and inexpensive vaccine production [71]. Therefore, VV vectors have been examined for use as live vaccines against both human and veterinary infectious diseases and cancers [6,7,8]. However, concerns about the safety profile of VVs are a major barrier to developing recombinant VV vaccines for use in humans [72].

Most research has focused on replication-defective poxvirus vectors (which have better safety profiles) as vehicles for delivering antigens derived from human pathogens. For example avipox- [73], MVA- and NYVAC-based vectors expressing components of human pathogens, such as HIV-1 and tuberculosis, have been developed and evaluated in monkeys [74,75] and humans [76,77,78,79]. However, although promising in animal models, these vaccines did not induce sufficiently strong immune responses in humans, nor did they protect humans from infection [79,80]. Therefore, more effective vehicles are needed for human vaccine development.

Thus, a replication-competent VV that has been proven to be safe for human vaccination against smallpox could be a good candidate. The safety profile and strong antigenicity of LC16m8∆, a genetically-stable variant of LC16m8, make it a promising vehicle for a vaccine against HIV or other human diseases.

One concern regarding the use of viral vectors is pre-existing immunity against the vector virus, which has the potential to dampen specific immune responses. However, Kohara *et al.* showed that a recombinant LC16m8 vaccine expressing the SARS coronavirus (SARS-CoV) spike protein elicited neutralizing antibodies against SARS-CoV in rabbits that generated a high titer of anti-LC16m8 antibodies [81]. Another report shows that the VV lacking the B5 ectodomain induces a more potent immune response in vaccinia-immune animals than its wild-type counterpart [82]. These results suggest that LC16m8∆ would make a good vector virus for eliciting effective immune responses against foreign antigens in individuals pre-immunized with smallpox vaccines.

Previously, we developed the pSFJ1-10 promoter, an A-type inclusion body (ATI) complex promoter that comprises ten repeat units of the mutated early region of the p7.5 promoter plus the ATI late promoter [83]. This complex promoter possesses strong activity in both the early and late phases of the VV infection cycle. Indeed, the H protein of the measles virus and chloramphenicol acetyltransferase, the synthesis of which is driven by this promoter, comprised approximately 10% of total cellular protein [84,85]. Moreover, we constructed LC16m8∆VNC110, a vector that harbors pSFJ1-10 along with a multiple cloning site within the hemagglutinin (HA) gene, which can be used for the rapid production of recombinant LC16m8∆ through *in vitro* ligation of the LC16m8∆VNC110 genome with foreign DNA [86]. The foreign genes inserted were stably maintained in the LC16m8∆ recombinants constructed by this technique and harboring the p7.5 promoter after several passages in the RK13 cells, a standard cell line for the propagation of VVs.

Using this technique, we tested whether LC16m8∆ is a better vector than non-replicating vaccinia virus for the expression of SIV Gag. The Gag proteins of HIV-1 and SIV are major antigens that elicit cytotoxic T lymphocyte (CTL) responses. The activity of anti-Gag CTL inversely correlates with the viral load in HIV-1-infected individuals [87]. Experimental infection of monkeys with SIV suggests that the strength of the anti-Gag CTL response correlates with the containment of SIV [88]. A m8∆/pSFJ/SIVGag vector expressing the SIV Gag antigen under control of the pSFJ1-10 promoter generated significantly more Gag protein *in vitro* and elicited the production of anti-Gag IFN-γ^+^ T-cells in mice, more efficiently than the non-replicating VV DIs strain (which harbors the *gag* gene under the control of the same promoter) [86]. The DIs strain is immunogenically similar to MVA [89].

We further optimized LC16m8∆ for use as a vector by comparing the immunogenicity of SIV Gag proteins expressed under the control of either the pSFJ1-10 promoter or the p7.5 promoter, which is a classical early-late promoter [90] with moderate activity (although weaker than that of pSFJ1-10). Preliminarily observations indicated that expressing too much foreign protein led to a reduction in VV propagation *in vitro*; therefore, the balance between the expression of a foreign antigen and viral propagation *in vivo* might be crucial for optimal immunogenicity. Thus, we compared the immunogenicity and virulence of m8∆/p7.5/SIVGag with that of m8∆/pSFJ/SIVGag in the setting of a recombinant Bacillus Calmette-Guerin (BCG) prime/recombinant LC16m8∆ boost vaccination protocol. This setting was based on the observation that long-term maintenance of effector memory T-cells (Tem) with the capacity to immediately attack SIV-infected cells restricts infection by antibody-resistant SIV at the site of virus entry. This was achieved using vaccine approaches that persistently express viral antigens in vaccinated macaques via the use of a cytomegalovirus (CMV) vector, thereby resulting in continuous immune stimulation [91,92]. Since BCG persists in vaccinated individuals for long periods of time (up to 10 years) without serious symptoms, vaccination with BCG expressing the Gag protein may be expected to induce Gag-specific CD8^+^ T-cells and to maintain immunological memory (via Tem) for a long time. Vaccination studies in mice revealed that m8∆/pSFJ/SIVGag was less pathogenic and elicited Gag-specific IFN-γ^+^, CD107α^+^ and CD8^+^ T-cells more efficiently than m8∆/p7.5/SIVGag. Tem were detected even at four months after boosting with m8∆/pSFJ/SIVGag. Therefore, LC16m8∆ that express SIV Gag under the control of the pSFJ1-10 promoter induced more efficient and long-lasting immune responses than LC16m8∆ harboring the p7.5 promoter [93].

Although inducing both anti-HIV-1 antibody and cytotoxic CD8^+^ T-cells is an effective way of preventing HIV-1 infection, it is often difficult to induce the production of anti-HIV-1 antibodies, particularly neutralizing antibodies, at a high titer. For example, only a low titer of anti-HIV-1 Env antibodies was observed, even after repeated immunization with an MVA-based vector [94]. Repetitive antigenic stimulation is required for affinity maturation, the process by which high avidity neutralizing antibodies against HIV-1 are generated. Long-lasting expression of antigen by a replication-competent vector, such as LC16m8∆, may enable repeated immunological presentation, which induces affinity maturation.

We next examined the ability of LC16m8∆ expressing the HIV-1 Env gene to elicit anti-HIV-1 antibodies and CD8^+^ T-cells in mice in the setting of a recombinant LC16m8∆ prime followed by a Sendai virus vector boost. We found that this vaccination regimen led to the efficient induction of both Env-specific CD8^+^ T-cells and anti-Env antibodies, including neutralizing antibodies. These results are in sharp contrast to those reported by studies that used vaccine regimens based on priming with an Env-expressing plasmid followed by a boost with the LC16m8∆ or SeV vector; such an approach mainly induced cell-mediated immune responses [95].

## 5. Conclusions

Despite its replication-competent phenotype, LC16m8∆ is highly attenuated and shows no pathogenic effects in SCID mice (similar to replication-defective VVs, such as MVA). However, it is a comparably effective smallpox vaccine with respect to Dryvax. Moreover, LC16m8∆-based vectors induce both antibody- and cell-mediated immune responses against foreign antigens more efficiently than non-replicating VV vectors. Therefore, LC16m8∆ is superior to non-replicating VV vectors and is suitable for use in humans. We also point out that LC16m8∆ recombinants may be useful as a dual vaccine against both smallpox and pathogens targeted with the inserted genes.
